# Community’s misconception about COVID-19 and its associated factors among Gondar town residents, Northwest Ethiopia

**DOI:** 10.1186/s41182-020-00279-8

**Published:** 2020-12-07

**Authors:** Habtamu Sewunet Mekonnen, Abere Woretaw Azagew, Chalachew Adugna Wubneh, Getaneh Mulualem Belay, Nega Tezera Assimamaw, Chilot Desta Agegnehu, Telake Azale, Zelalem Nigussie Azene, Mehari Woldemariam Merid, Atalay Goshu Muluneh, Demiss Mulatu Geberu, Getahun Molla Kassa, Melaku Kindie Yenit, Sewbesew Yitayih Tilahun, Kassahun Alemu Gelaye, Animut Tagele Tamiru, Bayew Kelkay Rade, Eden Bishaw Taye, Asefa Adimasu Taddese, Zewudu Andualem, Henok Dagne, Kiros Terefe Gashaye, Gebisa Guyasa Kabito, Tesfaye Hambisa Mekonnen, Sintayehu Daba Wami, Jember Azanaw, Tsegaye Adane, Mekuriaw Alemayehu

**Affiliations:** 1 Department of Medical Nursing, School of Nursing, College of Medicine and Health Sciences, University of Gondar, Gondar, Ethiopia; 2 Department of Pediatrics and Child Health Nursing, School of Nursing, College of Medicine and Health Sciences, University of Gondar, Gondar, Ethiopia; 3 School of Nursing, College of Medicine and Health Sciences and Comprehensive Specialized Hospital, University of Gondar, Gondar, Ethiopia; 4 Department of Health Education and Behavioral Sciences, Institute of Public Health, College of Medicine and Health Sciences, University of Gondar, Gondar, Ethiopia; 5 Department of Women’s and Family Health, School of Midwifery, College of Medicine and Health Sciences, University of Gondar, Gondar, Ethiopia; 6 Department of Epidemiology and Biostatistics, Institute of Public Health, College of Medicine and Health Sciences, University of Gondar, Gondar, Ethiopia; 7 Department of Health Systems and Policy, Institute of Public Health, College of Medicine and Health Sciences, University of Gondar, Gondar, Ethiopia; 8 Departmnet of Psychiatry, College of Medicine and Health Sciences, University of Gondar, Gondar, Ethiopia; 9 Department of General Midwifery, School of Midwifery, College of Medicine and Health Sciences, University of Gondar, Gondar, Ethiopia; 10 Department of Clinical Midwifery, School of Midwifery, College of Medicine and Health Sciences, University of Gondar, Gondar, Ethiopia; 11 Department of Environmental and Occupational Health and Safety, Institute of Public Health, College of Medicine and Health Sciences, University of Gondar, Gondar, Ethiopia; 12 Department of Gynecology and Obstetrics, University of Gondar College of Medicine and Health Sciences, Gondar, Ethiopia

**Keywords:** Misconception, COVID-19, Gondar city, Northwest Ethiopia

## Abstract

**Background:**

Despite the implementation of various strategies such as the declaration of COVID-19 emergency state, staying at home, lockdown, and massive protective equipment distribution, still COVID-19 is increasing alarmingly. Therefore, the study aimed to assess the community’s perception of COVID-19 and its associated factors in Gondar town, Northwest Ethiopia.

**Methods:**

A community-based cross-sectional study was employed among 635 Gondar administrative town residents, from April 20 to April 27, 2020. Study participants were selected using a cluster sampling technique. Data were collected using an interviewer-administered structured questionnaire. Epi-Data version 4.6 and STATA 14 were used for data entry and analysis, respectively. Logistic regressions (bivariable and multivariable) were performed to identify statistically significant variables at *p* < 0.05.

**Results:**

Of the 635 study participants, 623 have completed the study with a 98.1% response rate. The mean age of participants was 36.32 years (SD ± 13.24). The overall magnitude of the community’s misconception about COVID-19 stood at 56.9% (349). Age and religion showed a negative association with misconceptions. To be specific, being in the age group of 27–33 (AOR = 0.52, 95% CI 0.32, 0.86) and being a Muslim (AOR 0.51, 95% CI 0.34, 0.78) were negatively associated with the misconception of COVID-19, whereas occupation and awareness showed positive associations with the misconception. To be specific, having an unemployed occupational status (AOR = 1.79, 95% CI 1.14, 2.82) and being unaware of the number of cases of COVID-19 (AOR 1.66, 95% CI 1.05, 2.62) were positively associated with the community’s misconception on COVID-19.

**Conclusion:**

The magnitude of the community’s misconception about COVID-19 among Gondar town residents was high. Age, religion, unemployment, and unawareness about the number of COVID-19 cases were significant factors of misconception about COVID-19. Thus, stakeholders ought to build community perceptions about COVID 19. To resolve misinformation about COVID-19, accurate and relevant information should be provided to the community using appropriate communication media.

## Background

Coronavirus diseases 19 (COVID-19) is defined as an illness caused by a novel coronavirus now called severe acute respiratory syndrome coronavirus 2 (SARS-COV-2; formerly called 2019 novel coronavirus or 2019-nCov) [1, 2]. The virus was first identified on December 30, 2019, in Wuhan city, Hubei province, China. It is highly contagious, causing flu-like symptoms, and spreads worldwide which became a global pandemic [3].

The clinical spectrum of COVID-19 varies from being asymptomatic to severe clinical conditions like respiratory failure [4]. The virus can enter the body from the reservoirs mainly through respiratory droplets during coughing, sneezing, and touching. Wearing a face mask, keeping social distance more than 3 ft, and staying at home are the strategies implemented to break this outbreak [3]. Nevertheless, weak health system, equipment shortage, lack of robust containment measures, problem in managing mobility, economic vulnerability, limited fiscal spacing [5], and the unknown biologic nature of the disease [6] are the main challenges in COVID-19 pandemic prevention.

The virus can survive in different environment, temperature, and humidity conditions [7]. Even though COVID-19 affects all people, its risk is high among people with hypertension, diabetes mellitus, chronic obstructive pulmonary diseases, cardiovascular diseases, cerebrovascular disease, and other co-morbidities [8]. As studies showed, of the total coronavirus cases, about 80% displayed mild symptoms, and 14% developed severe complications like pneumonia. Only 5% have been critical, and 1% were asymptomatic [9, 10].

COVID-19 can result in complications such as thrombocytopenia [11], sepsis-induced coagulopathy [12], acute encephalopathy [13], systemic inflammation, multiorgan dysfunction, acute myocardial infarction, lung and heart failure, and dysrhythmias [14]. Thus, COVID-19 has become the cause of a massive global health crisis [15] that affects the social, mental, and psychological well-being of the world’s population [16].

There is a lot of information out there about coronavirus, but not all are true [17]. Most people believed that COVID-19 is a stigmatized disease despite the efforts of COVID risk communication and public education [18].

Misconception can be present in different levels of a community. Some people believed that wearing a surgical mask is most effective, and eating from a Chinese restaurant is highly risky to acquire the virus [19]. A study in Jeddah, Saudi Arabia, recorded a misconception rate of 66.9% [16]. In Nigeria, 83% of participants of a study held at least one misconception related to COVID-19 at which they believed that the virus originated in the laboratory. Accordingly, misconceptions have consequences on the short- and long-term control efforts against the disease, and it is one of the health hazards in the prevention of coronavirus [20].

Policy and health education modifications are essential for minimizing the adverse health effects of COVID-19 [21]. Community engagement is also of paramount importance in joint prevention and control of confronting uncertainty and countering rumors effectively [22]. Focusing on measures such as campaigns to raise awareness, disprove myths, and induce compliance is vital in preventing the spread of COVID-19 [10]. Even though there is an implementation of different strategies such as declaring COVID-19 emergency state, staying at home, lockdown, and massive protective equipment distribution, the COVID-19 cases are increasing alarmingly from time to time [7]. Therefore, the study aimed to assess the community perception toward COVID-19 and identify the factors associated with it.

## Method and materials

### Study design and period

A community-based cross-sectional study design was employed from April 20 to 27, 2020.

### Study area

The study was conducted at the selected kebeles of Gondar town. The town of Gondar is located at 750-km distance, northwest of Addis Ababa, the capital city of Ethiopia. According to the 2015 population projection of major cities in Ethiopia, the total population of Gondar town was estimated to be 323,900. The town has 22 kebeles. Currently, it has one referral hospital and eight government health centers.

### Population

All people above 18 years of age in Gondar town and those in the selected kebeles were taken as source and study population, respectively.

### Sample size and sampling technique

The sample size is determined using a single population proportion formula by considering the following statistical assumptions:

Confidence level (Cl) of 95%

Proportion = 50%

Margin of error of 5%

Using the following single proportion formula:

*n* = $$ \frac{\left( Za/2\right)2\times P\left(1-P\right)}{(W)2} $$where:

*n* = initial sample size

*Z* = 1.96, the corresponding *Z*-score for the 95% CI

*P* = proportion = 50%

*W* = margin of error = 5% = 0.05

*n* = $$ \frac{\left(\mathbf{1}.\mathbf{96}\right)\mathbf{2}\times \mathbf{0.5}\left(\mathbf{1}-\mathbf{0.5}\right)}{\left(\mathbf{0.05}\right)\mathbf{2}} $$ = 384

By considering a 10% non-response rate, and a design effect of 1.5, the final sample size was 635. Finally, participants’ households were accessed using a cluster sampling technique.

From a total of 22 kebeles, eight kebeles (kebele 7, kebele 8, kebele 9, kebele 13, kebele 16, kebele 17, kebele 18, kebele 20) were selected by using a lottery method. Then, from each kebele, one to two Ketenas (the lowest administrative cluster) were selected, depending on the number of households. Those selected Ketenas were considered clusters, and all households in the selected Ketenas were involved, and one of the parents in each household was interviewed. A family member aged 18 years and above was the respondent whenever the parents were not available at the time of data collection.

### Data collection tools and procedures

Data regarding the socio-demographic, information exposure, risk perception, and precaution measure adoption and misconception were collected through face-to-face interviews using a structured questionnaire adapted from different literature. Respondents were asked the sources of information about COVID-19 and how much they trusted those sources. Respondents were also asked about the types of information that they wanted to receive. Participants were asked whether they were taking precautionary measures like avoiding handshaking, adopting hand washing, and practicing physical distancing.

The data were collected by 24 BSc nurses, and the overall data collection process was strictly followed by 6 supervisors. A 1-day training was given to the data collectors and supervisors about the purpose of the study, data collection tools, collection techniques, and ethical issues during the selection of participants and collection of the data. All answers to close- and open-ended questions were written down manually by the interviewers. Daily, the supervisors assessed the consistency and completeness of the data.

### Operational definitions

#### Misconception

In this study, 11 questions were developed to assess the community’s misconception of COVID-19. Each of the questions had five possible response categories ranging from strongly disagree (1) to strongly agree (5), which gives a possible total individual score of 55. First, negatively and positively constructed questions were recoded to the response categories. Then, individual and overall scores were computed. Since the data did not have a normal distribution, the median score was used to determine who had misconceptions and who did not have a misconception.

Finally, participants who had a median and above score of the COVID-19 misconception assessment questions were coded as having misconceptions otherwise no misconceptions.

#### Information exposure

Respondents were asked whether they have heard about various aspects of COVID-19, and the responses were coded “yes” or “no.”

#### Knowledge level

After data cleaning, the knowledge questions were recoded to the respective values, and the overall result was computed. Since the knowledge data did not have a normal distribution, the median score was used to determine the knowledge level. Accordingly, participants who had a median and above score of the knowledge questions about COVID-19 were labeled as having good knowledge otherwise poor knowledge.

#### Attitude

The attitude questions were recoded to the respective values, and the overall result was computed. Since the attitude data did not have a normal distribution, the median score was used to determine the attitude level. Accordingly, participants who had a median and above score of the attitude questions about the COVID-19 and its preventive measures were labeled as having a favorable attitude otherwise unfavorable attitude.

### Study variables

#### Dependent variable

- ✓Community’s misconception about COVID-19

#### Independent variables

- ✓Socio-demographic variables
- ✓Information exposure level
- ✓Knowledge about COVID-19
- ✓Attitude toward COVID-19 and its preventive measures
- ✓Self-perceived health status
- ✓Perceived dangerousness of COVID-19
- ✓Worry about COVID-19

### Statistical analysis

The data entry was performed using the statistical program Epi-Data version 4.6 and then exported into STATA 14 for analysis. Descriptive statistics were carried out and presented with narration, tabulation, and graphical presentation. Binary logistic regression (bivariable and multivariable) was performed so as to identify statistically significant variables. Variables with a *p* value < 0.2 in the bivariable analysis were candidate variables for multivariable logistic regression. To declare statistically significant variables, the adjusted odds ratio with a 95% confidence interval and a *p* value < 0.05 were used in the multivariable analysis. The Hosmer-Lemeshow goodness of fit test was performed, and the decision was made at a *p* value > 0.05.

### Quality assurance mechanisms

To assure the quality of the data, the tool was prepared first in English and then translated into Amharic (the local language in the study area) by language experts. Data collectors and supervisors were trained on the data collection process for 1 day. The data collection tool was pretested on 5% of the total sample size in sub-cities which had not been selected for actual data collection. Modifications were made to the questionnaire accordingly. Data collectors were closely monitored by investigators and supervisors. Moreover, the data quality was assured by using statistical parameters for assessing the validity of the collected data.

## Result

### Socio-demographic and personal characteristics of respondents

Out of the 635 selected study participants, 623 agreed to participate in the study, making up a response rate of 98.1%. Among the participants, 174 (27.9%) were in the age group of 34–45 years, with the mean age of 36.32 years (± 13.24 standard deviation). Four hundred two (64.5%) were female, and 373 (59.9%) of the participants were married. Four hundred thirty-three (69.5%) were Orthodox Christians. Two hundred two (32.4%) had at least a college education, and 448 (72%) were unemployed. More than half (55.2%) of the participants had a household of 4–6 occupants. The majority (90.5%) of the participants had good perceived health status, and 91.5% perceived COVID-19 as dangerous. Two thirds (71.3%) were worried about the COVID-19 (Table 1).

**Table 1:** Socio-demographic and personal characteristics of the study participants among Gondar town residents, Northwest Ethiopia, 2020 (*n* = 623)

Variables	Frequency (***n***)	Percent
Age (in years)		
18–26	163	26.2
27–33	150	24.1
34–45	174	27.9
> 45	136	21.8
Sex		
Male	221	35.5
Female	402	64.5
Current marital status		
Unmarried	250	40.1
Married	373	59.9
Religion		
Orthodox	433	69.5
Muslim	154	24.7
Protestant	27	4.3
Others	9	1.5
Educational status		
No formal education	125	20.1
Primary education	101	16.2
Secondary education	195	31.3
College and above	202	32.4
Occupation		
Unemployed	448	72
Employed	175	28
Household size		
1–3	178	28.6
4–6	344	55.2
7 and above	101	16.2
Self-perceived health status		
Good	564	90.5
Bad	59	9.5
Perceived dangerousness of COVID-19		
Dangerous	570	91.5
Like the common cold/flu	53	8.5
Worry about COVID-19		
Worried	444	71.3
Not worried	109	17.5
Worried as it is common cold/flu	70	11.2

### Knowledge, attitude, and information exposure of respondents about COVID-19

In this study, half (50.7%) of the participants had good knowledge about COVID-19. About 57.5% and 52% of participants had favorable attitudes toward COVID-19 and its preventive measures, respectively. The majority, 97.1%, 84.7%, 84.6%, and 81.7% of participants had heard about prevention, symptoms, transmission, and complications of COVID-19, respectively (Table 2).

**Table 2:** Knowledge, attitude, and information exposure of study participants about COVID-19 among Gondar town residents, Northwest Ethiopia, 2020 (*n* = 623)

Variables	Frequency	Percent
Knowledge about COVID-19		
Poor knowledge	307	49.3
Good knowledge	316	50.7
Attitude toward COVID-19		
Unfavorable attitude	265	42.5
Favorable attitude	358	57.5
Attitude toward prevention measures of COVID-19		
Unfavorable attitude	229	48
Favorable attitude	324	52
Heard about prevention methods of COVID-19		
Yes	605	97.1
No	18	2.9
Heard about COVID-19 symptoms		
Yes	528	84.7
No	95	15.3
Heard about COVID-19 transmission		
Yes	527	84.6
No	96	15.4
Heard about COVID-19 distribution		
Yes	269	43.2
No	354	56.8
Heard about the number of COVID-19-infected people		
Yes	293	47.0
No	330	53.0
Heard about the intervention of COVID-19 by the government		
Yes	248	39.8
No	375	60.2
Heard about the actions if someone infected by COVID-19		
Yes	184	29.5
No	439	70.5
Heard about the complication of COVID-19		
Yes	114	18.3
No	509	81.7

### Community’s misconceptions about COVID-19

In this study, the overall magnitude of the community’s misconception about COVID-19 was found to be 56.9% (95% CI 52–59). Of all participants, 44.3% and 36.3% responded “agree” for questions on COVID-19 mainly affects older people and it is easy to become infected with COVID-19 on an airplane, respectively. About 30.5% and 41.7% of the participants responded “disagree” for questions it is safe to receive packages from foreigners and wearing a face mask is enough to protect oneself from catching COVID-19, respectively. Regarding the question whether a hot cup of coffee or tea kills the coronavirus, 39.5% of the participants responded “agree” (Table 3).

**Table 3:** Magnitude of COVID-19 misconceptions among Gondar town residents, Northwest Ethiopia, 2020 (*n* = 623)

Variables	Strongly disagree	Disagree	Neutral	Agree	Strongly agree
Do you agree COVID-19 mainly affects older people?	44 (7.1%)	61 (9.8%)	14 (2.3%)	276 (44.3%)	228 (36.6%)
Do you believe that it is easy to become infected with COVID-19 on an airplane?	54 (8.7%)	135 (21.7%)	113 (18.1%)	226 (36.3%)	95 (15.25%)
Do you believe that it is safe to receive packages from foreigners?	88 (14.1%)	190 (30.5%)	103 (16.5%)	170 (27.3%)	72 (11.6%)
Do you think that wearing a face mask is enough to protect you from catching COVID-19?	123 (19.7%)	260 (41.7%)	45 (7.2%)	134 (21.5%)	61 (9.8%)
A hot cup of coffee or tea will help kill the virus.	109 (17.5%)	173 (27.8%)	47 (7.5%)	246 (39.5%)	48 (7.7%)
Eating garlic and onions will help ward off the virus.	121 (19.4%)	201 (32.3%)	38 (6.1%)	208 (33.4%)	55 (8.8%)
Drinking alcohol does not protect you against COVID-19.	90 (14.5%)	140 (22.5%)	50 (8.0%)	205 (32.9%)	138 (22.2%)
COVID-19 virus can be transmitted in areas with hot and humid climates.	95 (15.3%)	168 (27.0%)	72 (11.6%)	213 (34.2%)	75 (12.0%)
COVID-19 does not affect Africans.	239 (38.4%)	232 (37.2%)	47 (7.5%)	80 (12.8%)	25 (4.0%)
Spray alcohol and chlorine all over your body to protect against COVID-19.	93 (14.9%)	217 (34.8%)	62 (10.0%)	199 (31.9%)	52 (8.4%)
Adding pepper to your soup or other meals does prevent or cure COVID-19.	117 (18.8%)	201 (32.3%)	45 (7.2%)	220 (35.3%)	40 (6.4%)

### Factors associated with the community’s misconceptions of COVID-19

In the bivariable logistic regression analysis, age, religion, educational status, occupation, information about COVID-19 symptoms, information about COVID-19 distribution, and information about the number of COVID-19 were significantly associated factors.

However, in the multivariable logistic regression, age, religion, occupation, and information about the number of COVID-19-infected people remained significantly associated with the community’s misconception of COVID-19.

Participants in the age group of 27–33 years were 52% (AOR = 0.52, 95% CI 0.32, 0.86) less likely to have a misconception of COVID-19 compared to their younger age group (18–26 years of age). The misconception of COVID-19 was 51% (AOR 0.51, 95% CI 0.34, 0.78) less likely among Muslim participants compared to Orthodox Christian participants.

Unemployed participants were 1.79 (AOR = 1.79, 95% CI 1.14, 2.82) times more likely to have a misconception about the COVID-19 compared with the employed participants. Participants who never heard about the number of COVID-19-infected people were 1.66 (AOR 1.66, 95% CI 1.05, 2.62) times more likely to have misconceptions of COVID-19 compared to those participants who had heard about the numbers of COVID-19-infected people (Table 4).

**Table 4:** Bivariable and multivariable logistic regression analysis of factors for misconceptions of COVID-19 among Gondar town residents, Northwest Ethiopia, 2020 (*n* = 623)

Variables	Misconception of COVID-19		COR, 95% CI	AOR, 95% CI
	No	Yes		
Age (in years)				
18–26	72	91	1	1
27–33	88	62	0.56 (0.36, 0.87)	0.52 (0.32, 0.86)**
34–45	72	102	1.12 (0.73, 1.73)	1.06 (0.66, 1.71)
> 45	42	94	1.77 (1.10, 2.25)	1.67 (0.92, 3.03)
Sex				
Male	104	117	1	1
Female	170	232	1.21 (0.87, 1.69)	1.11 (0.77, 1.61)
Religion				
Orthodox	176	257	1	1
Muslim	79	75	0.65 (0.45, 0.94)	0.51 (0.34, 0.78)**
Protestant	14	13	0.64 (0.29, 1.39)	0.61 (0.26, 1.42)
Others	5	4	0.55 (0.15, 2.07)	0.53 (0.13, 2.27)
Educational status				
No formal education	36	89	2.38 (1.48, 3.82)	1.06 (0.53, 2.12)
Primary education	48	53	1.06 (0.66, 1.71)	0.71 (0.39, 1.31)
Secondary education	91	104	1.10 (0.74, 1.63)	0.76 (0.47, 1.25)
College and above	99	103	1	1
Occupation				
Unemployed	180	268	1.73 (1.22, 2.46)	1.79 (1.14, 2.82)**
Employed	94	81	1	1
Self-perceived health status				
Good	252	312	1	1
Bad	22	37	1.36 (0.78, 2.36)	0.72 (0.38, 1.38)
Perceived dangerousness of COVID-19				
Dangerous	257	313	1	1
Like a common cold/flu	17	36	1.74 (0.95, 3.17)	1.91 (0.96, 3.82)
Worry about COVID-19				
Worried	204	240	1	1
Not worried	46	63	1.16 (0.76, 1.78)	1.19 (0.73, 1.97)
Worried as it is common cold/flu	24	46	1.63 (0.96, 2.76)	1.35 (0.74, 2.48)
Knowledge about COVID-19				
Poor knowledge	127	180	1.23 (0.90, 1.69)	1.01 (0.67, 1.51)
Good knowledge	147	169	1	1
Attitude toward COVID-19				
Unfavorable attitude	106	159	1.32 (0.96, 1.83)	1.14 (0.80, 1.63)
Favorable attitude	168	190	1	1
Attitude toward prevention measures of COVID-19				
Unfavorable attitude	123	176	1.23 (0.91, 1.72)	1.23 (0.88, 1.78)
Favorable attitude	151	173	1	1
Heard about COVID-19 symptoms				
Yes	243	285	1	1
No	31	64	1.76 (1.12, 2.80)	1.43 (0.84, 2.45)
Heard about COVID-19 distribution				
Yes	139	130	1	1
No	135	219	0.55 (0.23, 0.87)	1.44 (0.92, 2.26)
Heard about the number of COVID-19-infected people				
Yes	153	140	1	1
No	121	209	0.64 (0.31, 0.96)	1.66 (1.05, 2.62)*

*Significant at *p* value < 0.05

**Significant at *p* value < 0.01 of adjusted odds ratio

## Discussion

Nowadays, COVID-19 is a global concern for discussion among the media as well as the public. Its mode of transmission has also induced tension among people around the world. But in low-income countries like Ethiopia, awareness of the community about COVID-19 varies in different settings. For this reason, we investigated the community’s misconception about COVID-19 and its associated factors among Gondar town residents.

The magnitude of community misperception about COVID-19 among the Gondar community was found to be 56.9% (95% CI 52, 59). About 42.5% of the study participants have an unfavorable attitude toward COVID 19. However, 97.1% and 84.7% had heard about prevention and transmission methods of COVID-19, respectively, whereas about 53% of the study participants did not hear about the number of infected persons in the Gondar community.

The finding of the current study about the magnitude of misconception about COVID-19 is lower than that of the Saudi population study [16]. The discrepancy could be due to the method of data collection, socio-demographic characteristics of the participants, and measurement tools. The researchers of the current study have designed an interview-based questionnaire so that the community might correctly answer. In case of accessing data online, the outcome might be inflated. Besides, the time difference between the two studies might also be one possible reason. The current study was conducted after the disease distribution increased globally, and awareness of the population increased relatively. This may reduce the misconception toward COVID-19.

Participants in the age group of 27–33 years were 52% less likely to have misconceptions of COVID-19 compared to those in the age group of 18–26 years. Older adults were more sensitive to get accurate knowledge about COVID-19, and they had difficulty in implementing the prevention mechanisms (hand washing, physical distancing, and wearing masks) of COVID-19, for they had fear regarding the nature of the pandemic. Alongside the severity of the COVID-19, the illness became worse in older ages which made the older adults dig out more information to realize the COVID-19 pandemic prevention [23]. Older adults have more information acquisition compared to lower age groups [24]. Furthermore, the population in this age group may feel responsible to protect themselves and others from the pandemic than the younger age group [25]. For this reason, they may search for accurate information that reduces the misconception toward the disease.

Occupation is one of the factors associated with the misconception toward COVID-19. Unemployed participants were 1.79 more likely to have a misconception about COVID-19 compared to participants with employed occupational status. This result has been supported by a study done in Saudi [16]. People who were unemployed mostly have lower education status compared with employed ones. These could burden the unemployed people by the inflow of information from different sources like Facebook and other sources, and they would be confused and worried about the finding of accurate knowledge [26, 27].

The misconception about COVID-19 was 51% less likely among Muslim participants compared to Orthodox Christian participants. In this case, Muslim followers can practice their religion independently. An individual Muslim can perform his/her religious practice in his/her house, which helps to reduce physical contact. On the other hand, in Orthodox Christianity, most of the services are provided by the priests, i.e., to get spiritual services, everyone should go to the church, which could contribute to physical contact. Besides, Christian followers believe that the COVID-19 is a result of punishment from God or is the work of the devil. So, they believe that this pandemic disease would be eliminated by the help of God. As a result, they may trust more information from religious leaders, and they may ignore information from health authorities which may lead to the misconception about the pandemic [28]. Therefore, most of the people ignored some principles like physical distancing and others.

Participants who had not heard of the number of COVID-19-infected people were 1.66 times more likely to have misconceptions about COVID-19 compared to their counterparts. Most of the time, people may underestimate health-promoting behaviors like hand washing and overestimate harmful behaviors. So, providing accurate information about what most people are doing is very important in health promotion. When people heard about the number of COVID-19 infection, it may give emphasis and empower themselves to know about the preventive measures, symptoms, and mode of transmission of the pandemic virus.

Therefore, people who had a positive knowledge about COVID-19 could be instrumental in reducing the disease because they can spread positive interventions like hand washing and physical distancing by demonstrating them to a wide range of people [29].

### Limitations

This study does not show a temporal relationship. The study may have a social desirability bias. In addition, the study was limited to the town and its surrounding, which may not be representative of the rural area which has problems related to access to information.

## Conclusion

The magnitude of the community’s misconception about COVID-19 among Gondar town residents was high. Age, religion, unemployment, and unawareness about the number of COVID-19-infected people were significant factors of misconception about COVID-19 among the residents. Besides, COVID-19 has an impact on social, economic, and political issues. Thus, stakeholders need to promote positive community perceptions about COVID-19. To resolve misinformation about COVID-19, accurate and relevant information should be provided to the community using appropriate communication media.

## Data Availability

The summary data are available in the main document, and the dataset analyzed is available from the corresponding author on reasonable requests.

## Abbreviations

AOR: Adjusted odds ratio
CI: Confidence interval
COVID-19: Coronavirus disease 2019
COR: Crude odds ratio
OR: Odds ratio
SD: Standard deviation
SPSS: Statistical Package for Social Sciences
WHO: World Health Organization
